# Removal of boron from aqueous solution using magnetic carbon nanotube improved with tartaric acid

**DOI:** 10.1186/2052-336X-12-3

**Published:** 2014-01-06

**Authors:** Nima Zohdi, Fariba Mahdavi, Luqman Chuah Abdullah, Thomas SY Choong

**Affiliations:** 1 Department of Chemical and Environmental Engineering, Universiti Putra Malaysia, Serdang, Selangor, Malaysia; 2 Advanced Materials and Nanotechnology Laboratory, Institute of advanced technology, Universiti Putra Malaysia, Serdang, Selangor, Malaysia

**Keywords:** Adsorption, Multi-walled carbon nanotube, Tartaric acid, Modification, Boron removal, Magnetic

## Abstract

Boron removal capacity of multi-walled carbon nanotubes (MWCNTs) modified with tartaric acid was investigated in this study. Modification of MWCNTs with tartaric acid was confirmed by Boehm surface chemistry method and fourier transform infra-red (FT-IR) spectroscopy. Experiments were performed to determine the adsorption isotherm and adsorption thermodynamic parameters of boron adsorption on tartaric acid modified MWCNTs (TA-MWCNTs). The effect of variables including initial pH, dosage of adsorbent, contact time and temperature was investigated. Analysis of data showed that adsorption equilibrium could be better described by Freundlich isotherm and the maximum adsorption capacities obtained at the pH of 6.0 was 1.97 mg/g. The estimated thermodynamic values of free energy (ΔG°), entropy (ΔS°) and enthalpy (ΔH°) indicated a spontaneous and an endothermic process. Furthermore, the TA-MWCNTs was magnetized for separation of boron-contaminated adsorbent from aqueous solution by applying magnetic field. The results showed that magnetic TA-MWCNTs particles were separated effectively after adsorption from contaminated water.

## Introduction

Water with less impurities and contaminants is essential to the human’s life. In general, water pollution is the introduction of physical, chemical and biological substances into the water bodies that spoils the purity of water and it will cause hazardous effects on living species that consume it. Boron (B) is one of the elements that can cause the lethal in case of more than 640 mg/kg body weight oral intake according to the world health organization (WHO) report [1,2].

In aqueous solution, boron is normally present as borate anions B(OH)^-^_4_ and boric acid B(OH)_3._ The dominant form of inorganic boron in acidic aqueous systems is the un-dissociated boric acid. On the other hand polyborate anionic species including B_5_O_6_(OH)_4_^-^, B_3_O_3_(OH)_4_, B_3_O_3_(OH)_5_^2-^ and B_4_O_5_(OH)_4_^2-^ form in high concentration solutions (>0.025 mol/l) at a neutral to alkaline pH (pH 6 to 11) [3,4].

High boron concentrations can be found in wastewater of some industries including semiconductor, ceramic, pesticides, fire retardants, borosilicate glass, nuclear power and detergent manufacturers. Many investigations has been done for boron removal from water and wastewater by different methods such as coagulation [5], coprecipitation [6], adsorption, ion exchange using cation exchangers [7], solvent extraction [8], membrane operations [9] and adsorption [5].

For treatment of boron in aqueous solution through adsorption process, different materials have been used as adsorbent such as activated carbon (AC) [10], fly ash [11], resins [12], metal oxides [13], clay materials [14], and composite magnetic particles [15].

Multi-walled carbon nanotubes (MWCNTs) are known as an effective adsorbent for removing contaminants such as various metals and heavy metals [16], dyes [17] and organic materials [18] from water and wastewater. The most important characteristics of MWCNTs are large specific surface area, well developed mesoporous and hollow structure and light mass density which make it an efficient adsorbent of pollutant molecules. Furthermore, MWCNTs have the advantages of easy removal and regeneration after contaminant adsorption [19]. Although, many researches have studied the capability of MWCNTs as an adsorbent of aqueous solution pollutants, however, adsorption capacity of boron onto MWCNTs has not been investigated so far.

The oxygen content of MWCNTs influences the maximum adsorption capacity. The oxygen functional groups such as –OH, –C = O, and –COOH can be generated on the surface of MWCNTs through covalent and noncovalent modification. MWCNTs were modified under the oxidizing condition with different chemicals such as HNO_3_, H_2_O_2_, KMnO_4_, NaClO, KOH, NaOH and citric acid [20,21]. Recent investigations showed that by using organic weak acids such as citric acid (C_6_H_8_O_7_), the functionalities can be produced without the negative signs depicted by the use of inorganic strong acids beside eliminating the refluxing step during the functionalization [22]. Due to the mild and safe reaction which organic materials can have and also the abovementioned advantages of these materials comparing to inorganic acids, the focus of this investigation is on MWCNTs modification using organic acids like tartaric acid (C_4_H_6_O_6_).

The influence of some parameters such as initial solution pH, dosage of adsorbent, initial boron concentration, contact time and temperature on boron adsorption behavior of tartaric acid modified MWCNTs (TA-MWCNTs) was studied in this work. TA-MWCNTs were modified with iron oxide particles for further magnetic separation of contaminated adsorbent.

## Materials and methods

### Chemicals

Boron stock solution was prepared by adding 5.71 g of boric acid B(OH)_3_ (Systerm Co.) into 1000 ml double distilled water. Azomethine-H monosodium salt hydrate >95% was purchased from Sigma Aldrich and L(+) ascorbic acid, ammonium acetate, glacial acetic acid, acid disodium salt-dihydrate (EDTA), mercaptoacetic acid 98% were purchased from Systerm Co. MWCNTs (diameter of 20–40 nm and minimum purity of 90%) and L(+)-tartaric acid >99.7% (C_4_H_6_O_6_) were purchased from Hangzhou Dayang Chem Co. and Sigma Aldrich Co. respectively. Ammonium iron (II) sulfate hexahydrate (NH_4_)_2_Fe(SO_4_)_2_ · 6H_2_O and hydrazine hydrate (N_2_H_4_) were received from Systerm Co. and R&M Chemicals. All the reagents used in this study were analytical grade.

### Modification of MWCNTs with tartaric acid

To find the optimum amount of the tartaric acid for modification of MWCNTs and to obtain the highest functional groups loading, 10 g of MWCNTs was mixed with 50 ml of four different concentrations of aqueous solution of tartaric acid (0.5, 1, 1.5 and 2 M). The mixtures were subjected to ultrasonic bath for 15 min mixing and then left to be dried and forming a paste followed by keeping the samples in the furnace for 30 min at 300°C. It is worth to mention that because of the decomposition of the excess of tartaric acid after increasing the temperature up to 300°C, further filtration step and washing of tartaric acid modified MWCNTs was not required [21].

The LECO TruSpec CHNS-O elemental analyzer was used for preliminary comparison of the oxygen content and carbon content of MWCNTs and TA-MWCNTs samples. By comparing the oxygen contents, a preliminary evaluation of optimum required amount of tartaric acid was possible and the sample with the highest oxygen content was selected for further surface chemistry analysis and proceeding the entire work. The Boehm titration technique [23] and solid addition technique [24] were employed to study the surface chemistry and point of zero charge (pH_pzc_) for MWCNTs and TA-MWCNTs.

To investigate the formation of carboxylic and carbonyl functional groups on TA-MWCNTs, the FT-IR spectra were obtained for MWCNTs and TA-MWCNTs in KBr pellet form with AEM Thermo Nicolet FT-IR collected at a spectrum resolution of 4 cm^-1^ with 32 co-added scans over the range from 4,000 to 400 cm^-1^.

To evaluate the specific surface area of MWCNTs before and after modification, Brunauer Emmett Teller (BET) analysis was used. After subjecting the samples with nitrogen gas for 9 hours of operation at 290°C of outgas temperature, the Quantachrome AS1Win™ surface area analyzer was used for measuring the surface area.

Transmission electron microscopy (TEM, Philips HMG 400) was also used to characterize the microstructure of MWCNTs before and after modification.

### Preparation of magnetic TA-MWCNTs

To modify the TA-MWCNTs surface with iron oxide particles [25], 6 g of ammonium iron (II) sulphate hexahydrate was dissolved in 200 ml of distilled water and hydrazine hydrate (volume ratio of 3:1). Then, 2.5 g of TA-MWCNTs was added into the solution. The pH of the mixture was adjusted to 11–13 and then the mixture was sonicated and stirred vigorously for about 15 min. After sonication, the mixture was refluxed for 2 hours. Finally, magnetic TA-MWCNTs was washed several times with distilled water using a filtration system until the pH of the solution became neutral. Then the magnetic TA-MWCNTs was dried under vacuum at 65°C for 24 hours.

The prepared magnetized sample was characterized using X-ray diffraction technique (XRD) using a Philips PW 3710 type diffractometer and FT-IR (AEM Thermo Nicolet). Morphology and chemical composition of the samples were studied using Energy-dispersive X-ray spectroscopy (EDX), and the scanning electron microscopy (SEM) using SEM-EDX Hitachi S-3400 N.

### Adsorption experiments

The effect of pH on boron adsorption was investigated using boron stock solution (20 mg/l) with pHs varied from 2–11. A specific amount of 0.32 g/l of TA-MWCNTs was added to boron stock solutions. The samples were kept in the shaker for 3 days in the room temperature (25°C) to reach equilibrium. The boron uptake on TA-MWCNTs was measured using UV–vis spectrophotometer (Double beam, Halo DB 20S) through Azomethine H method [10,26].

To study the effect of adsorbent quantity on boron adsorption, 6 samples of boron stock solution (20 mg/l) with pH of 6 were prepared. The TA-MWCNTs in various concentrations (0.16 to 0.56 g/l) were added to boron stock solutions and kept at shaker at 25°C for 3 days. The boron adsorption of mixtures was then measured using UV/Vis spectrophotometer.

The contact time and initial boron concentration dependent experiment was carried out with different stock solutions of initial boron concentrations varied from 2 to 40 mg/L. The solution was mixed with 0.1 g TA-MWCNTs and kept in a shaker for 24 h at room temperature. The boron adsorption of samples was measured in different time intervals using UV/Vis spectrophotometer.

Thermodynamic of the study was investigated in three different temperatures of 303, 313 and 323 K. All effective parameters of experiment which were obtained from optimal conditions of previous parts of the study were used to adjust the condition in temperature dependent experiment.

Adsorption behavior of magnetic-MWCNTs was investigated using the same method under the optimal adsorption conditions evaluated in the experiments.

The amount adsorbed boron per unit mass of adsorbent at equilibrium was given as follows [27]:

(1) q=eCo−Ce×VW

Where q_e_ (mg/g) was equilibrium uptake, C_0_ and C_e_ (mg/l) denoted the initial and equilibrium concentrations of boron in aqueous solution, V was the total volume of the solution in liters and W was the mass of the adsorbent in grams.

## Results and discussion

### Characterization of MWCNTs and TA-MWCNTs

To find out the optimum concentration of tartaric acid, the oxygen content of TA-MWCNTs samples obtained from different amount of tartaric acid which was measured by CHNS-O analysis is shown in Table 1. By increasing the concentration of tartaric acid from 0.50 to 1.00 M an increase in oxygen content happened. A further increase of concentration of Tartaric acid to 2 M caused negligible changes in the oxygen content. It was concluded that using 1 M solution of tartaric acid resulted in the highest amount of oxygen containing functional groups. Therefore, TA-MWCNTs sample modified with 1 molar solution was used to proceed the entire work.

**Table 1:** Oxygen percentage for TA-MWCNTs modified with different concentrations of tartaric acid obtained from CHNS-O analysis

**Sample**	**Tartaric acid concentration (M)**	**Oxygen content (wt.%)**
TA-MWCNTs	0.50	34.791
	1.00	35.805
	1.50	34.703
	2.00	34.805
MWCNTs	-	0.021

To obtain a precise evaluation of functionalities on the TA-MWCNTs, results of boehm method is shown in Table 2. By comparing the values for MWCNTs and TA-MWCNTs it can be concluded that after modification, oxygen functionalities such as carboxyl groups and phenolic groups were generated on the surface of TA-MWCNTs whilst MWCNTs had negligible carboxyl and phenolic groups. Also, the higher amount of oxygen containing functional groups resulted in the dominance of acidic character in TA-MWCNTs comparing to MWCNTs [23]. Forming oxygen contained functional groups was also reported in previous works by modification of MWCNTs with citric acid [22,28]. Although, the chemistry of reaction between nanotubes and citric acid or tartaric acid at high temperatures is still unknown, it could be mentioned that functional groups formed on the surface of TA-MWCNTs can serve as attachment points in the construction of complexes between B(OH)_4_/B(OH)_4_^-^ and TA-MWCNTs surface [29,30].

**Table 2:** Surface chemistry of MWCNTs and TA-MWCNTs derived from boehm titration

**Sample name**	**Functional group**	**Value (mmol/g)**
	Carboxyl groups	0.001
	Phenolic	0.003
MWCNTs	Acidity	0.033
	Basicity	0.008
	Carboxyl groups	0.398
TA-MWCNTs	Phenolic	0.637
	Acidity	1.036
	Basicity	0.016

**Figure 1:**
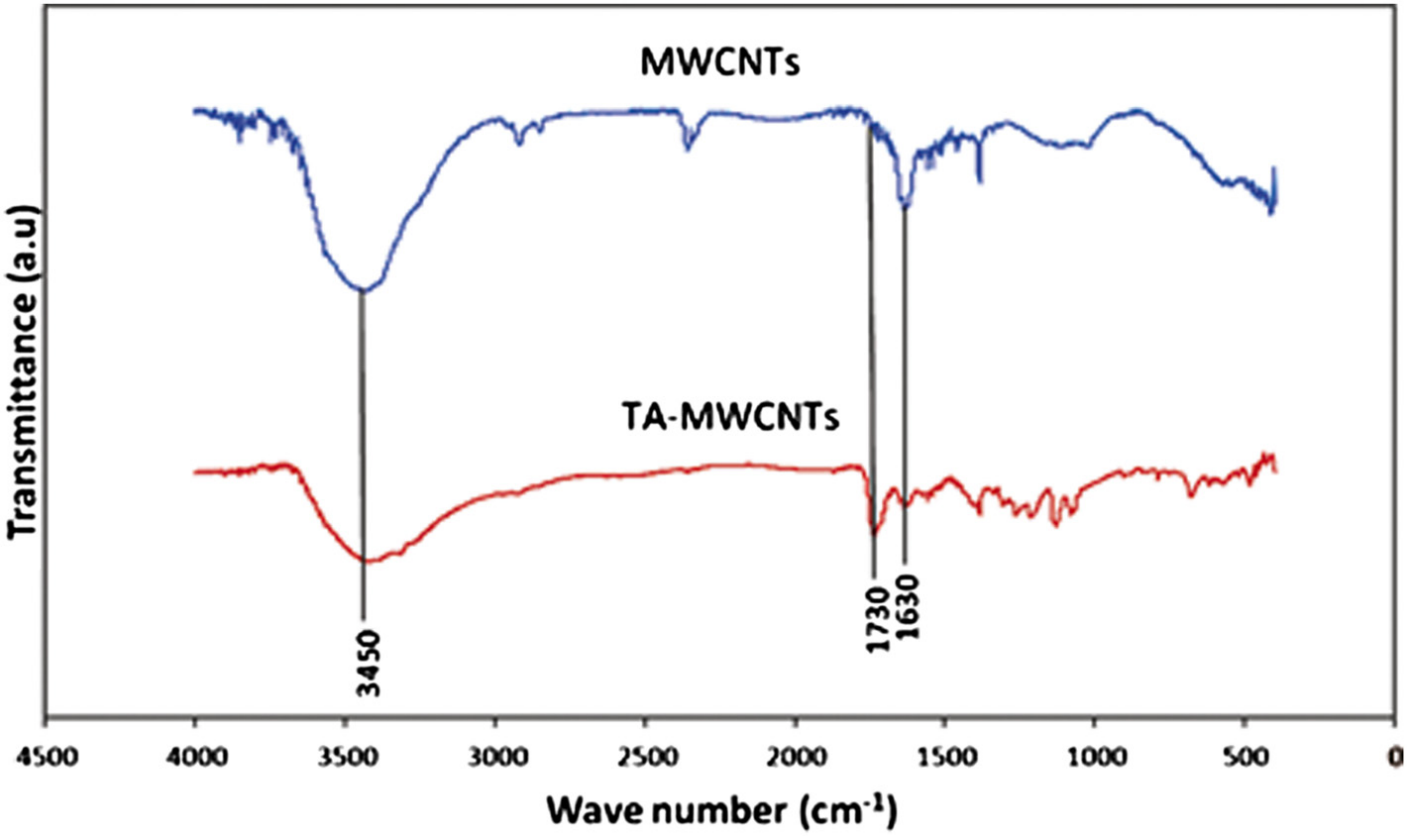
The FT-IR graph for MWCNTs and TA-MWCNTs.

The forming of functionalities was also proved by the comparison of the FT-IR spectra for MWCNTs and TA-MWCNTs (Figure 1). The FT-IR graphs for TA-MWCNTs and MWCNTs showed a broad peak at ~3425 cm^-1^ which is ascribed to the O–H stretching of the hydroxyl group. Also, the absorption peak at 1633(cm^-1^) was related to the C = C stretching mode of the TA-MWCNTs and MWCNTs [31]. The presence of carboxylic groups after the modification step was confirmed with a C = O band stretching that appeared at ~1730 cm^-1^ (−COOH). As it can be perceived from the graph, the C = O band was only appeared after the modification of MWCNTs with tartaric acid [32].

Table 3 presents some characteristics of MWCNTs before and after modification. The BET surface area for unmodified-MWCNTs was 99.93 m^2^/g. After modification the BET surface area of MWCNTs modified with tartaric acid decreased slightly to 99.61 m^2^/g. The decrease in BET surface area may be attributed to the partial blockage of pores by residual ashes of tartaric acid.

**Table 3:** Characteristics of MWCNTs and TA-MWCNTs

**Characteristics**	**Values for MWCNTs**	**Values for**
		**TA-MWCNTs**
BET	99.93 m^2^/cm	99.61 m^2^/cm
Particle size	Diameter: 30–50 nm	Diameter: 30–50 nm
pH_pzc_	7.40	6.20
Bulk density	0.26 g/cm^3^	0.28 g/cm^3^
Oxygen content	0.021%	35.805%
Carbon content	95.21%	98.60%

**Figure 2:**
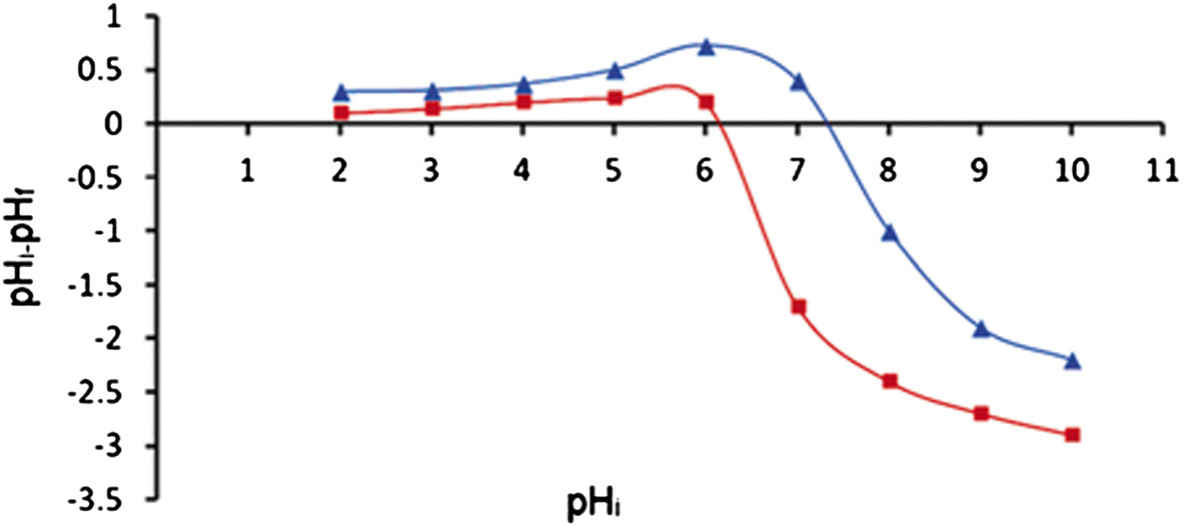
**pH**_**i **_**versus ∆pH for MWCNTs and TA-MWCNTs.**

Point of zero charge (pH_pzc_) for TA-MWCNTs and MWCNTs was calculated through the pH_i_ and pH_f_ values derived from solid addition technique. The point at which the ∆pH (pH_i_-pH_f_) equals zero would be considered as pH_pzc_. From Figure 2, the point of zero charge values for MWCNTs and TA-MWCNTs were determined to be around 7.40 and 6.20, respectively. The most obvious effect of forming oxygen functionalities (carboxylic and phenolic groups) is increase in surface acidity and consequently decrease in pH_pzc._ This result is in good agreement with the surface chemistry analysis.

**Figure 3:**
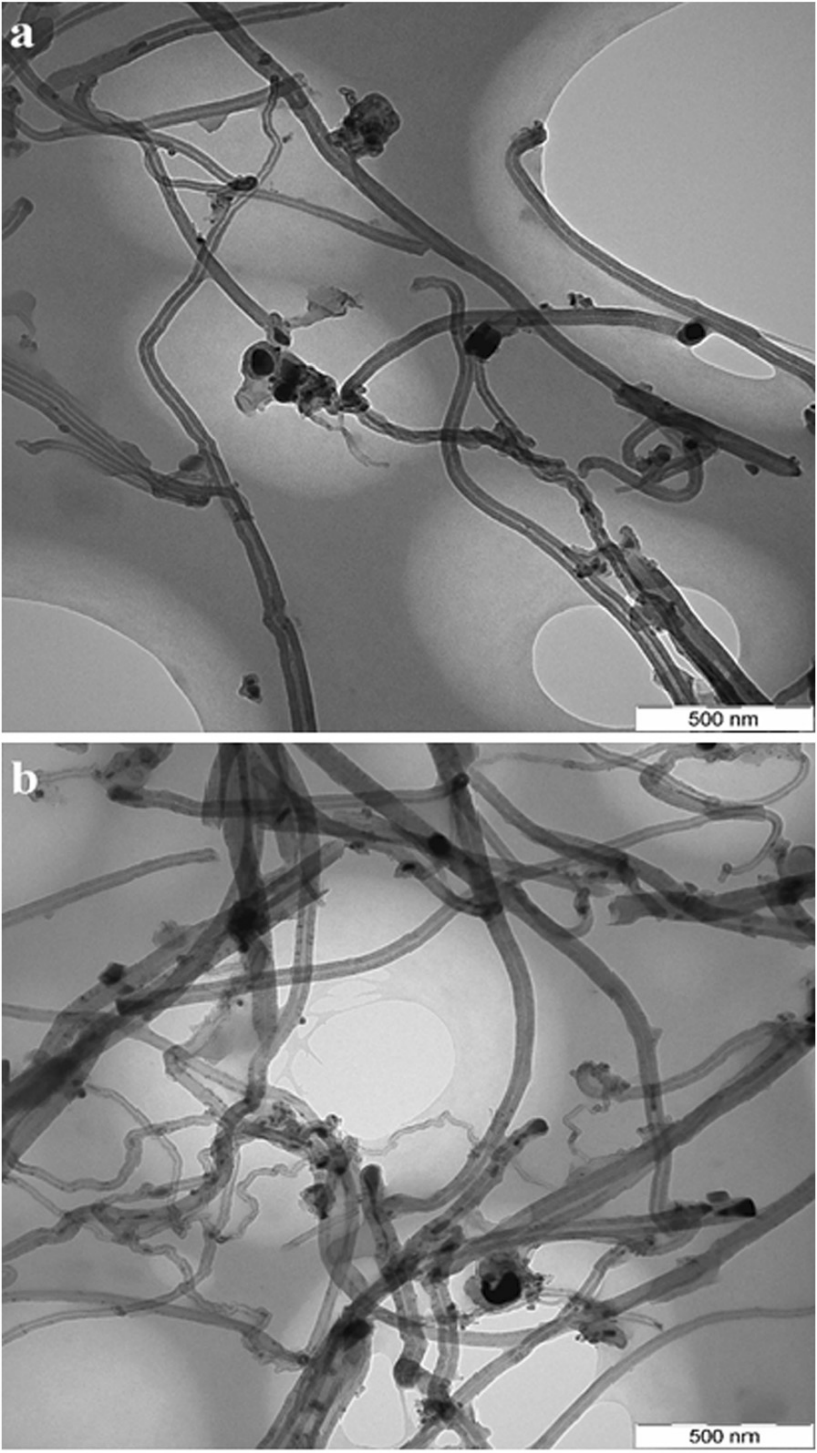
TEM image of a) MWCNT, b) TA-MWCNT.

TEM images of MWCNTs and TA-MWCNTs are shown in Figure 3a,b. Figure 3(a) shows the appearance of some black spots on MWCNTs which could be related to impurities. After modification of MWCNTs (Figure 3b) more black spots were formed on MWCNTs which is related to the residues of tartaric acid after decomposition at 300°C. Also, from the images the diameter of MWCNTs was estimated roughly in the range of 30–50 nanometers.

### Adsorption studies

#### Effect of initial solution pH

**Figure 4:**
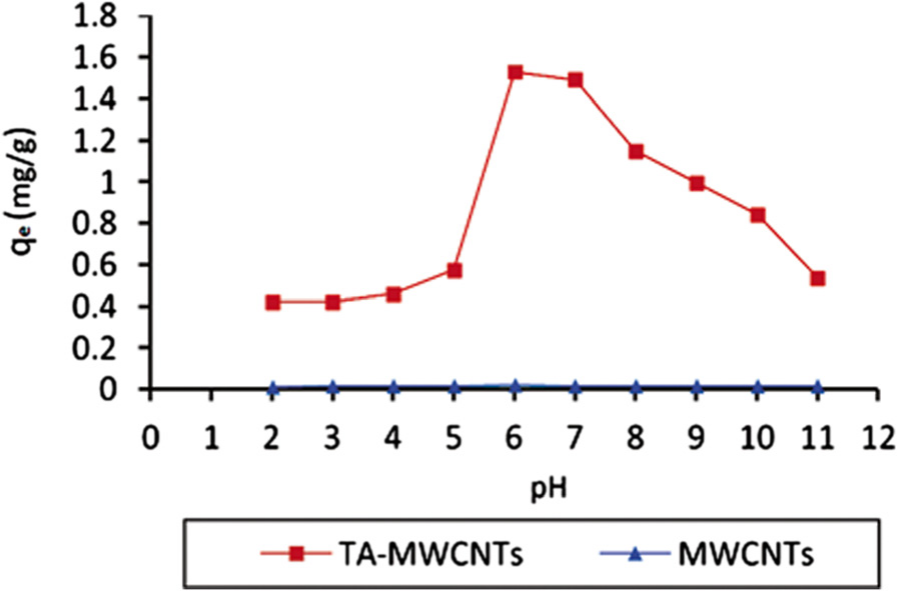
The effect of pH on boron adsorption on TA-MWCNTs and MWCNTs (dosage adsorbent: 0.32 g/l, boron concentration: 20 mg/l, temperature: 25°C).

Figure 4 shows the effect of initial solution pH on adsorption capacity of TA-MWCNTs and MWCNTs. MWCNTs showed negligible affinity (q_e_ = 0.01 mg/g) for all existing forms of boron in low and high pH values. On the other hand, by increasing the pH from 2 to 4, boron adsorption capacity of TA-MWCNTs was increased slightly from 0.42 mg/g to around 0.57 mg/g. At this range of pH (<pH_pzc_ of TA-MWCNTs), boron existed in the form of B(OH)_3_ and also the surface charge of TA-MWCNTs was positive. The slight increase in the affinity could be explained in terms of increasing the functional group which works as the sites of forming complex with boric acid. By increasing the pH from 5 to 6, the boron adsorption capacity reached to the maximum value of 1.53 mg/g. At pH of 6, the surface of TA-MWCNTs was positive, however besides existing the boron in form of B(OH)_3,_ the B(OH)_4_^-^ species with negative charges were formed and occurring electrostatic attraction made the highest affinity for boron. By increasing the pH from 6 to 11 (>pH_pzc_ of TA-MWCNTs) the gradual decrease in boron adsorption capacity could be assigned to the electrostatic repulsion between negative surface charge of TA-MWCNTs and negative charge of B(OH)_4_^-^ formed in high pHs.

### Effect of initial dosage of adsorbent

**Figure 5:**
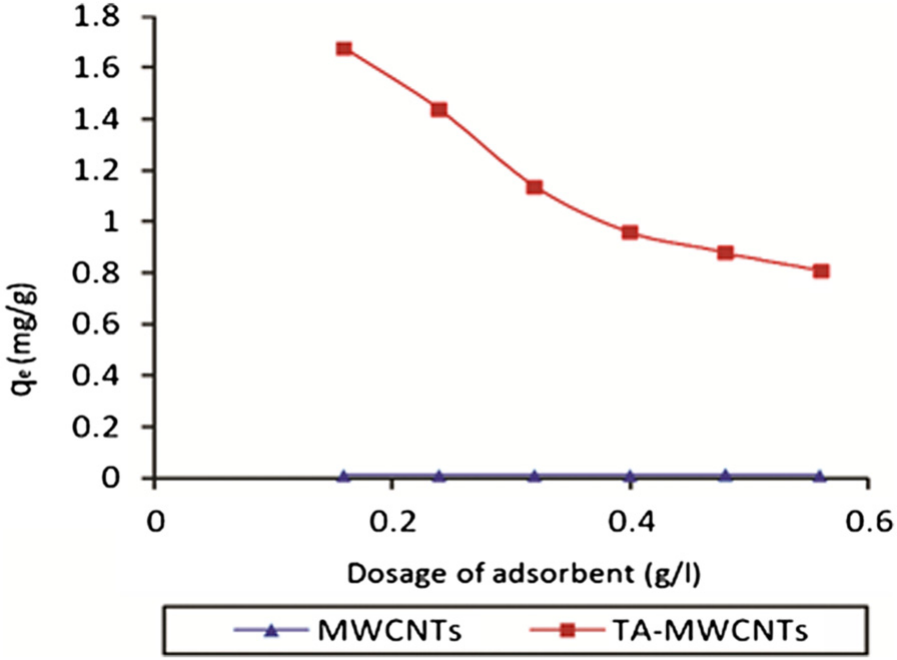
Effect of sorbent dosage on boron removal by MWCNTs and TA-MWCNTs (pH: 6, boron concentration: 20 mg/l, temperature: 25°C).

Figure 5 shows the effect of initial dosage of TA-MWCNTs on the adsorption capacity of sorbent. For TA-MWCNTs by increasing the adsorbent dosage, boron adsorption capacity decreased from 1.67 to 0.8 mg/g whereas the equilibrium point was happened at dosage adsorbent of around 0.4 g/l. The diminishing of adsorption capacity by increasing the sorbent dosage could be due to the fixed quantity of active adsorption sites of sorbent. These sites eventually became saturated with boron and therefore were unable to accommodate any more ions at higher concentrations. As was expected, by increasing the dosage of MWCNTs a very negligible changes in boron adsorption capacity (q_e_ = 0.01 mg/g) was observed.

### Effect of contact time and initial boron concentration

**Figure 6:**
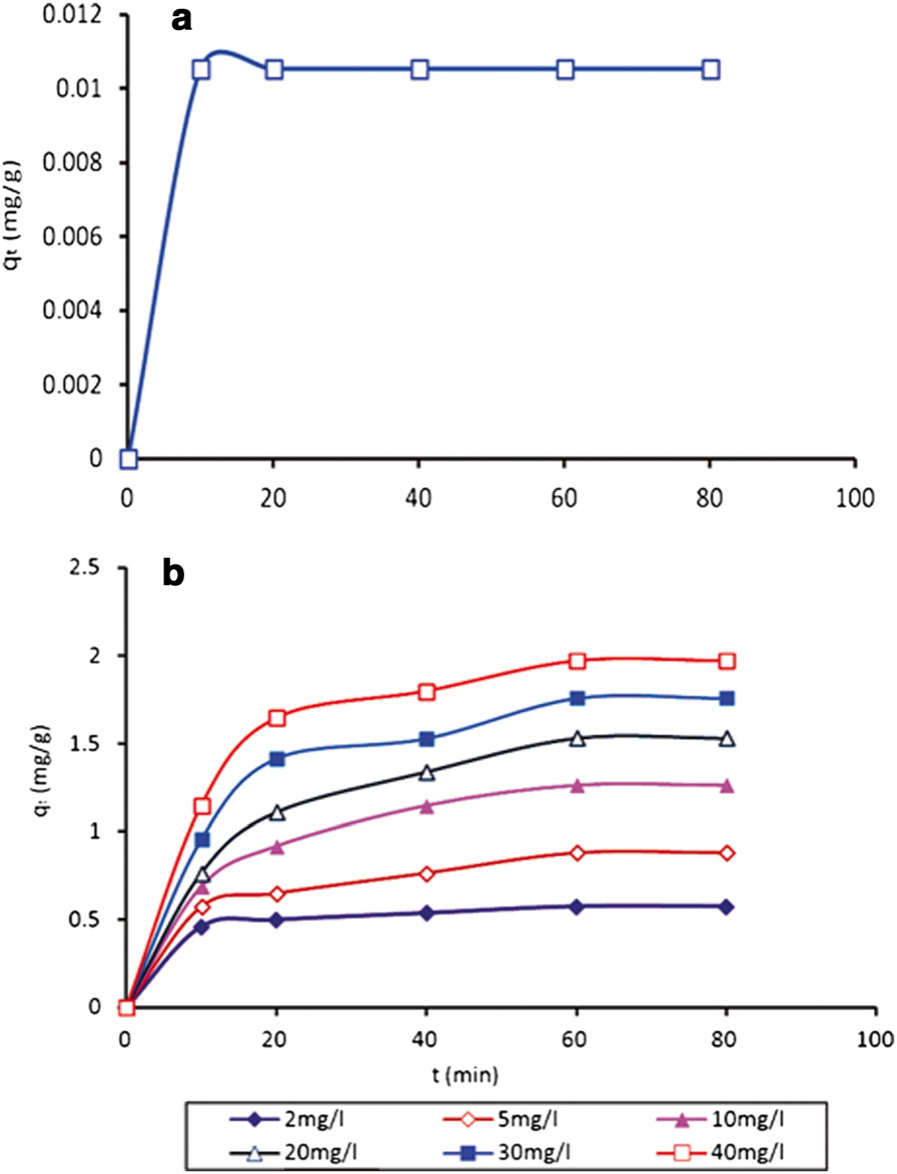
Effect of contact time and initial boron concentration on boron removal using MWCNTs (a) and TA-MWCNTs (b) (Temperature: 25°C, pH: 6, dosage adsorbent: 0.4 g/l).

The effect of contact time was investigated for unmodified MWCNTs and TA-MWCNTs by using synthetic solutions with initial concentration varied from 2 to 40 mg/l. As shown in Figure 6, the initial uptake of boron was rapid which could be attributed to the unsaturated surface of the TA-MWCNT. After that, the adsorption gradually slowed down by saturation of the surface and then became constant at around 60 minutes. The equilibrium adsorption capacity (*q*_*e*_) increased with increasing initial boron concentration.

For MWCNTs (Figure 6a), a constant boron adsorption capacity was recorded with increasing contact time of adsorbent and initial boron concentration. The small numbers of vacant sites on the surface of MWCNTs were occupied at the very initial stages and sorption capacity became constant in a very short duration.

For TA-MWCNTs (Figure 6b), the equilibrium point for all concentrations was around 60 minutes. The maximum adsorption happened at 40 mg/l of boron concentration and the amount was 1.97 mg/g.

From the results, it can be concluded that the optimum conditions of boron removal was achieved by using 0.4 g/l of TA-MWCNTs in temperature of 25°C, at pH of 6 and with 40 mg/l initial boron concentration after 60 min of contact time.

### Adsorption isotherm

The adsorption data obtained for TA-MWCNTs as a function of initial boron concentrations was used for finding the most appropriate linear forms of the Freundlich and Langmuir adsorption isotherms. The linear form of the Langmuir equation [33] is given as:

(2) $$\frac{C_{e}}{q_{e}}=\frac{1}{k_{l}}+\frac{a_{l}C_{e}}{k_{l}}$$

Where k_l_ (L/mg) and a_l_ (mg/L) are two Langmuir isotherm constants which are obtained from the intercept and slope of the linear plot of equation 2.

**Figure 7:**
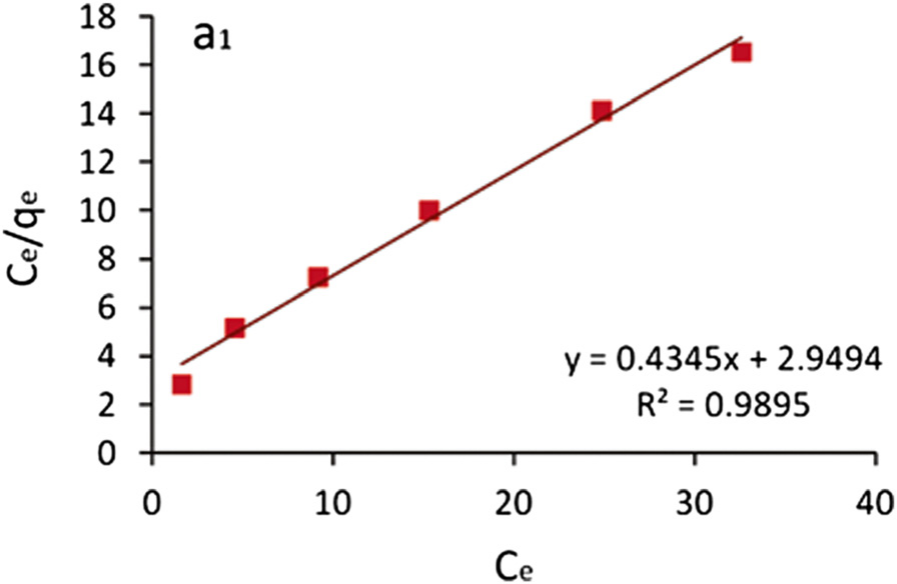
**Plot of C**_**e**_**/q**_**e **_**against C**_**e **_**for TA-MWCNTs.**

Figure 7 shows the plot of C_e_/q_e_ against C_e_ for TA-MWCNTs.

The data was also plotted using the linear form of the Freundlich equation [34]:

(3) $$q_{e}=K_{f}C_{e}{}^{1/n}$$

Where K_f_ is the Freundlich constant or capacity factor (mg/g) and 1/n is the Freundlich exponent.

**Figure 8:**
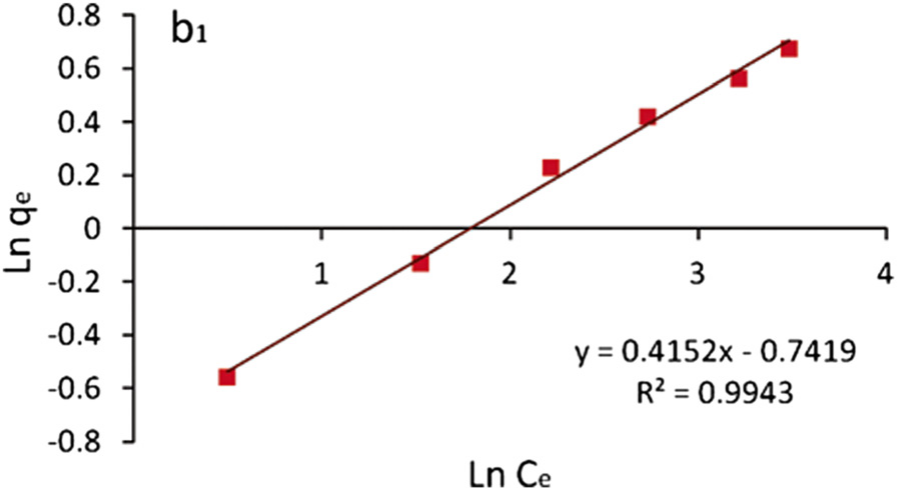
Plot of ln (qe) against ln (Ce) for TA-MWCNTs.

Figure 8 shows the plot of ln (*q*_e_) against ln (*C*_e_) for TA-MWCNTs. Figure 8 indicates clearly that TA-MWCNTs obeyed the Freundlich isotherm. The isotherm parameters are also presented in Table 4.

**Table 4:** Isotherm parameters for the adsorption of boron on TA-MWCNT

	**Freundlich constants**			**Langmuir constants**		
	**K**_**f **_**(mg/g)**	**n (mg/L)**	**R**^**2**^	**K**_**L **_**(L/g)**	**a**_**L **_**(mg/L)**	**R**^**2**^
Tartaric acid m-MWCNT	2.099	2.408	0.994	0.339	0.147	0.989

From the results presented in Figures 7 and 8 and Table 4, it can be concluded that the value of exponent n is in the range of desirable adsorption (1 < *n* < 10). The higher values of linear correlation coefficients (*R*^2^) for boron uptake (0.9943) revealed that the Freundlich model can be used to describe the boron adsorption behavior comparing to the Langmuir model.

**Figure 9:**
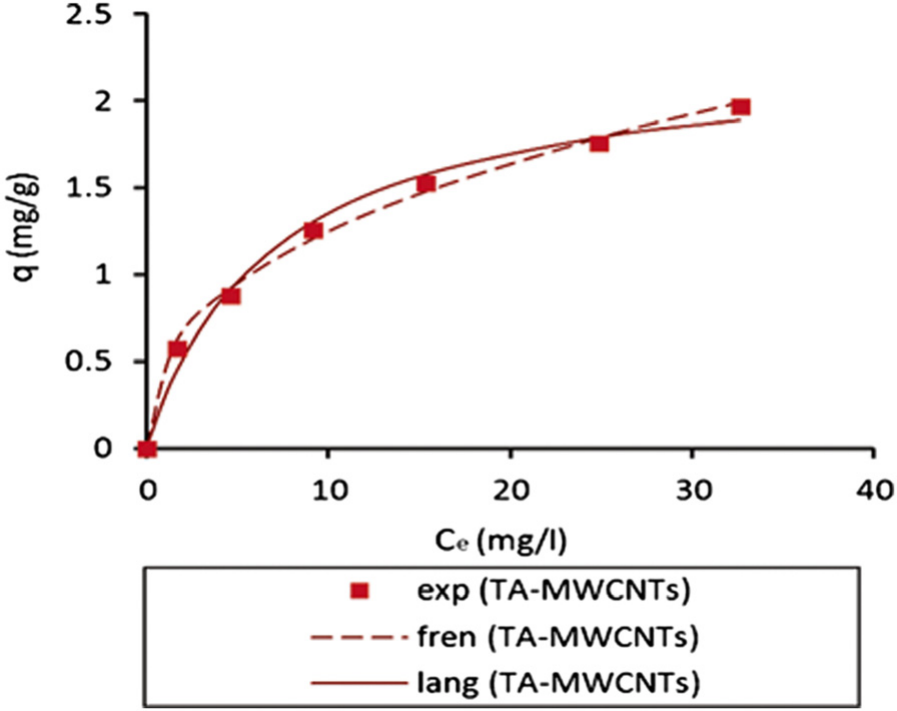
Comparison between experimental data and isothermal models.

Figure 9 compares the experimental results and isotherm models. It is concluded that the Freundlich isotherm fitted better to the experimental data comparing with the Langmuir model.

### Adsorption thermodynamics

To evaluate the effect of temperature, thermodynamic parameters consisting of standard free energy change (∆*G*^º^), standard enthalpy change (∆*H*^º^) and standard entropy change (∆*S*^º^) must be taken into consideration. The equilibrium constant (*K*_*c*_) was calculated by using the following equation [33]:

(4) $$K_{c}=\frac{C_{\mathrm{Ae}}}{C_{e}}$$

Where *C*_Ae_ is the amount of boron adsorbed (mmol/g), and *C*_e_ is the equilibrium concentration of boron in the solution (mmol/l).

Gibbs free energy changes (∆*G*^º^) was calculated using the following equation:

(5) $$\mathrm{\Delta G}{}^{\circ}=-\mathrm{RT}\ln K_{c}$$

Where absolute temperature is shown by *T* (K) and R is universal gas constant (8.314 J/mol-K). The values of enthalpy change (∆*H*^º^) and entropy change (∆*S*^º^) were calculated by using the Van’t Hoff equation as follows:

(6) $$\ln K_{c}=\frac{\mathrm{\Delta S}{}^{\circ}}{R}-\frac{\mathrm{\Delta H}{}^{\circ}}{\mathrm{RT}}$$

**Figure 10:**
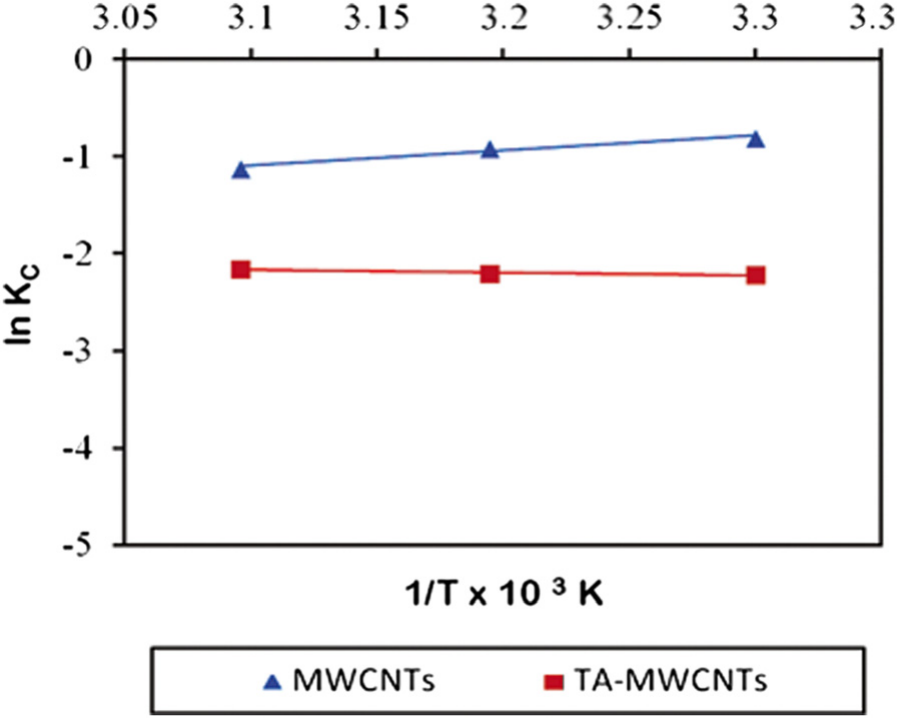
**Plot of ln (K**_**C**_**) versus (T**^**−1**^**) for estimation of thermodynamic parameters for adsorption of boron on MWCNTs and TA-MWCNTs.**

A plot of ln *K*c against 1/*T* rendered a straight line, as shown in Figure 10. The slope of the plot is equal to -ΔH° /R and its intercept value is equal to ∆*S*^º^ /R. These thermodynamic parameters are presented in Table 5.

**Table 5:** Thermodynamics parameters for boron adsorption on MWCNTs and TA-MWCNTs

**Temperature (k)**	**ln k**_**c**_	**∆G° (kJ/mol)**	**∆H° (kJ/mol)**	**∆S° (J/molK)**
MWCNTs				
303	−0.802	−2.022	12.719	48
313	−0.905	−2.356		
323	−1.116	−2.998		
TA-MWCNTs				
303	−2.279	−5.574	29.47	78.5
313	−1.828	−4.758		
323	−1.556	−4.181		

As presented in Table 5, the ∆G° values are negative and the ΔH° values are positive for TA-MWCNTs and MWCNTs. These results demonstrated that the adsorption of boron on MWCNTs and TA-MWCNTs was spontaneous and was an endothermic process. The decrease of negative values of ΔG° with the increase of temperature for MWCNTs indicated more efficient adsorption at higher temperature. On the other hand, for TA-MWCNTs since the negative values of ΔG° increased with increase in the temperature, the spontaneous adsorption process should be less efficient at higher temperatures.

### Characterization of magnetic TA-MWCNTs

**Figure 11:**
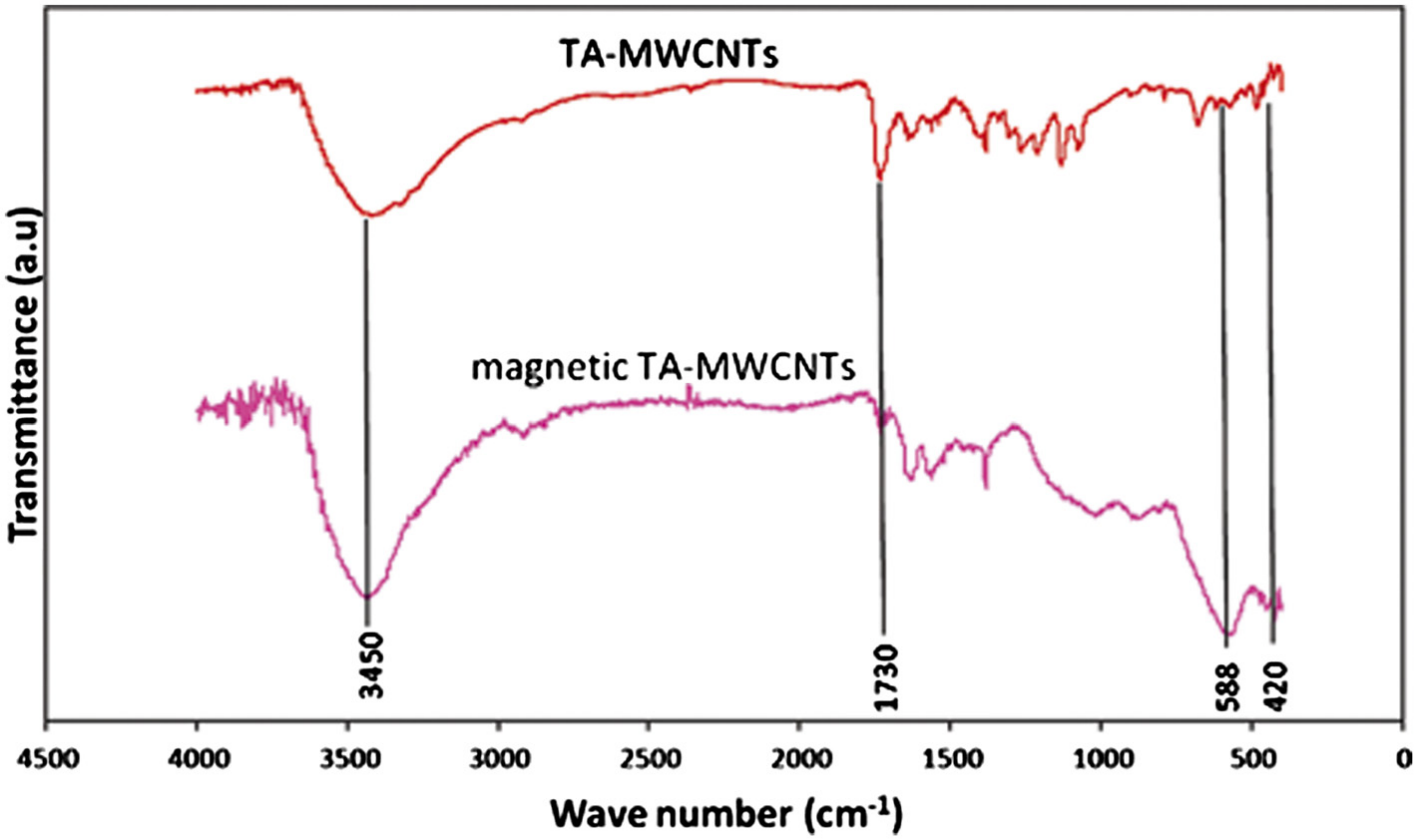
The FT-IR graph for TA-MWCNTs and magnetic TA-MWCNTs.

Figure 11 shows the FT-IR spectra for TA-MWCNTs before and after magnetizing. The peaks at 421 cm^-1^ and 588 cm^-1^ appeared after magnetizing the TA-MWCNTs. These peaks represented the existence of iron oxide particles on the surface of TA-MWCNTs [35]. Also, peaks at ~1729 and ~1627 indicated the existence of carboxylic groups on the surface of, TA-MWCNTs after magnetizing process.

**Figure 12:**
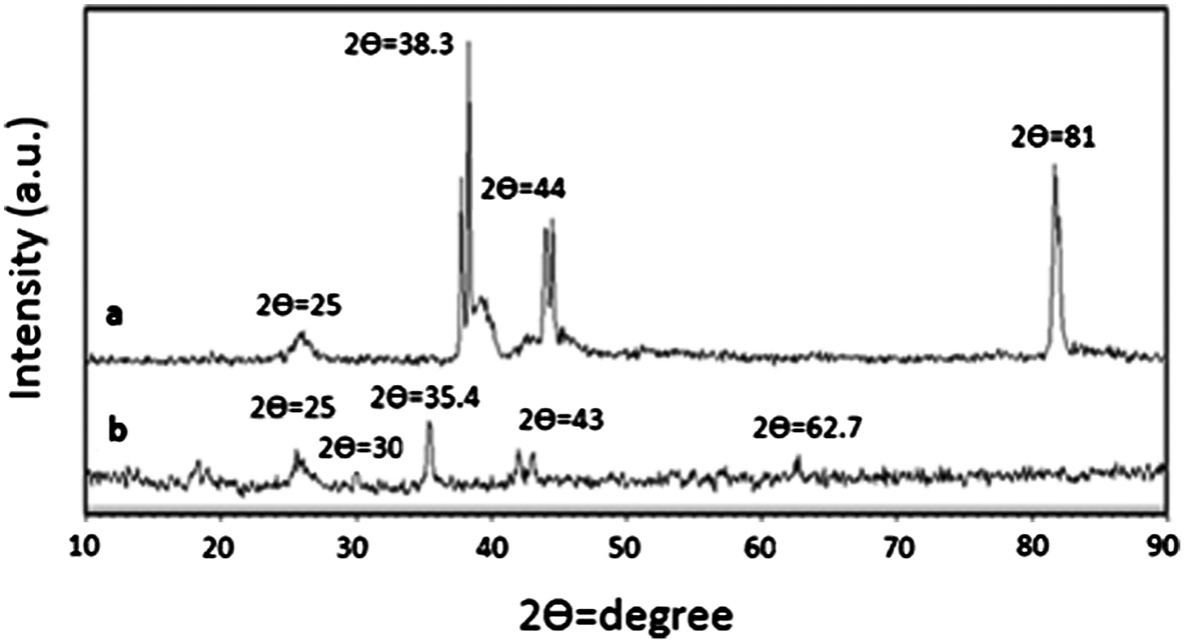
XRD patterns for (a) TA-MWCNTs and (b) magnetic TA-MWCNTs.

Figure 12 compares the XRD patterns for MWCNTs and TA-MWCNTs. Prevalently, four types of iron oxides forms under reaction conditions including magnetite (Fe_3_O_4_), maghemite (Fe_2_O_3_), hematite (γ-Fe_2_O_3_) and goethite (FeO(OH)), and among them, only magnetite and maghemite are magnetic. The XRD pattern of the magnetic TA-MWCNTs displayed three main diffraction peaks. The peaks at 2Ɵ = 35.4° and 43° (Figure 12b) are assigned to magnetite or maghemite. Other peaks are also observed at 2Ɵ = 30° and 62.7° for the magnetic TA-MWCNTs, which refer to the presence of hematite. The results had a good agreement with other reports and confirmed that iron oxide particles were formed on the surface of TA-MWCNTs [25,36]. It was also perceived that the typical peak (2Ɵ = 25°) corresponding to the MWCNTs (Figure 12a) also existed in the XRD pattern of the magnetic TA-MWCNTs (Figure 12b).

**Figure 13:**
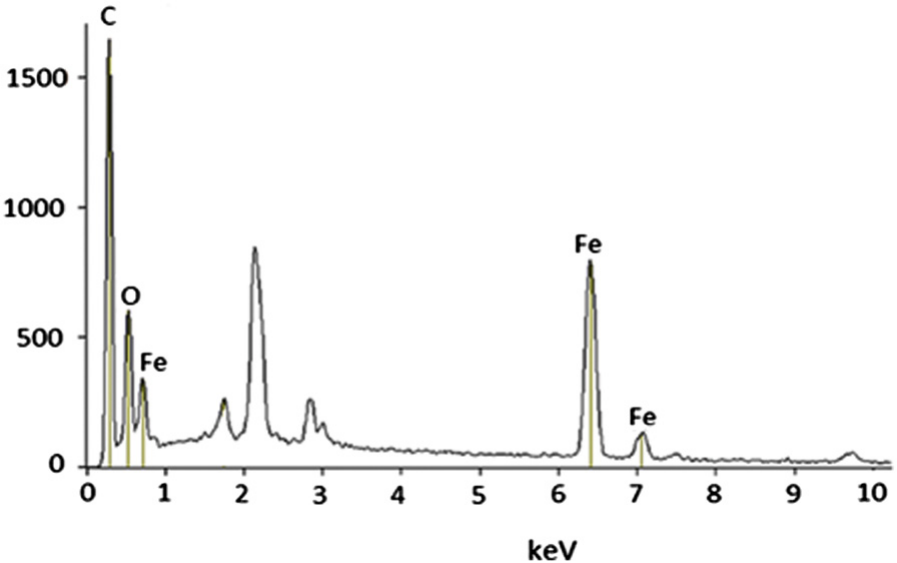
EDX spectrum for magnetic TA-MWCNTs.

Figure 13 shows the EDX results for magnetic TA-MWCNTs. The existence of iron and oxygen elements can be perceived by intense peaks on the surface of magnetic TA-MWCNTs.

**Figure 14:**
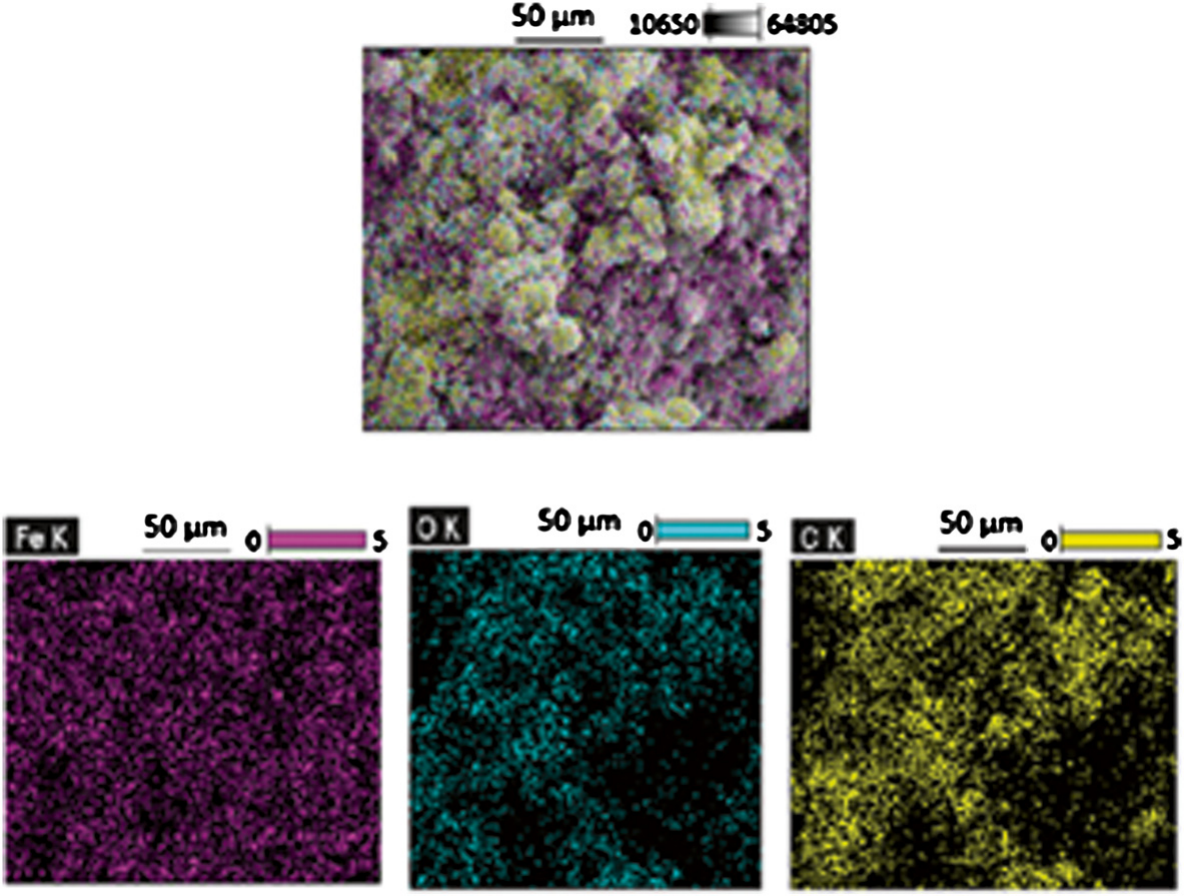
SEM-EDX mapping of magnetic TA-MWCNTs surface.

Figure 14 also shows the distribution of C, O, and Fe elements on the surface of magnetic TA-MWCNTs and confirmed the forming of magnetic iron oxide particles on TA-MWCNTs.

**Figure 15:**
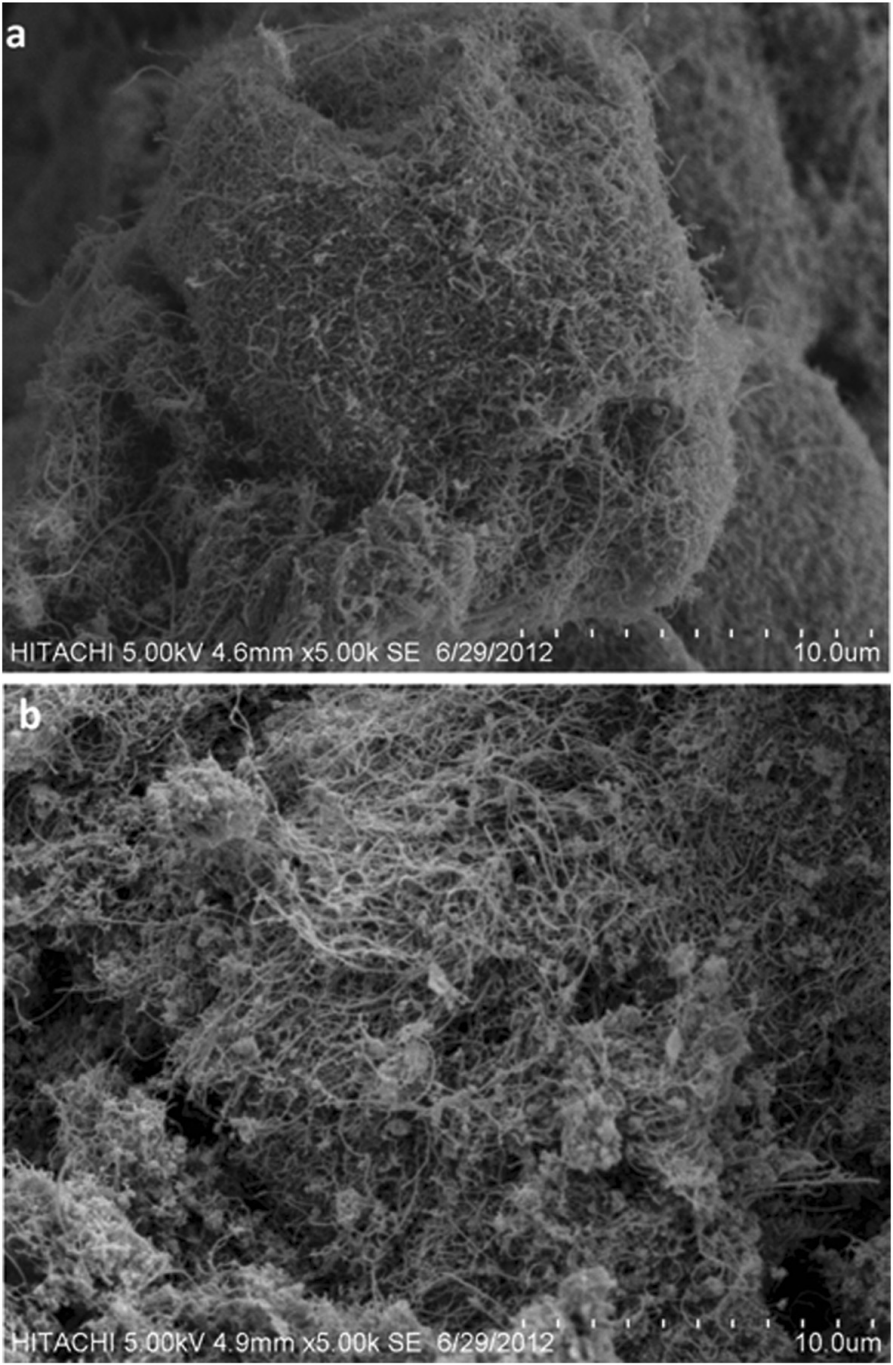
SEM images of (a) TA-MWCNTs and (b) magnetic TA-MWCNTs.

The SEM technique was used to investigate the morphology of MWCNTs before and after magnetizing. By comparing the images in Figure 15, it can be seen that an entangled network of TA-MWCNTs with clusters of iron oxides attached to them formed after magnetizing process. This indicated that iron oxides were successfully bounded on the surfaces of TA-MWCNTs to form magnetic TA-MWCNTs.

### Adsorption study of magnetic TA-MWCNTs

**Figure 16:**
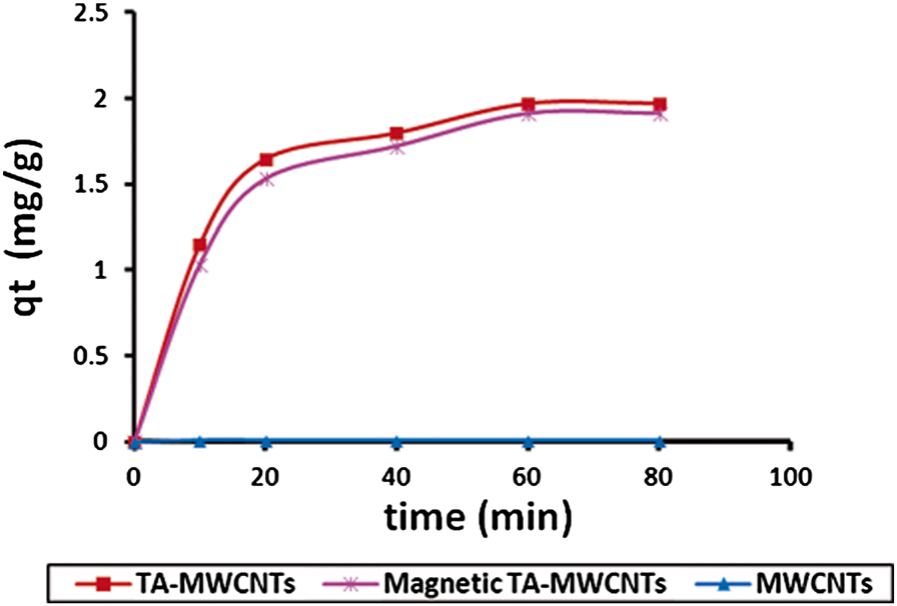
Boron adsorption capacity versus time for TA-MWCNTs, magnetic TA-MWCNTs and MWCNT at pH of 6, temperature of 25°C, contact time of 60 min, boron initial concentration of 40 mg/l and adsorbent dosage of 0.4 g/l.

The boron adsorption behavior of TA-MWCNTs after magnetizing was studied under optimal conditions (initial solution pH of 6, temperature of 25°C, contact time of 60 min, boron initial concentration of 40 mg/l, initial dosage of adsorbent of 0.40 g/l). Figure 16 compares the boron adsorption capacity for MWCNTs, TA-MWCNTs and magnetic TA-MWCNTs.

From the results after 60 min at equilibrium time boron adsorption capacity of magnetic TA-MWCNTs was measured to be 1.91 mg/g. However, by comparing the q_e_ values for TA-MWCNTs (1.97 mg/g) and magnetic MWCNTs it is perceived that a negligible decrease in boron adsorption capacity of TA-MWCNTs occurred after magnetizing. The decrease in boron adsorption capacity could be related to the occlusion of mesopores of TA-MWCNTs by iron oxide particles which led to less bonding of boron with oxygen functionalities anchored within the mesopores.

### Separation of contaminated magnetic TA-MWCNTs from aqueous solution

**Figure 17:**
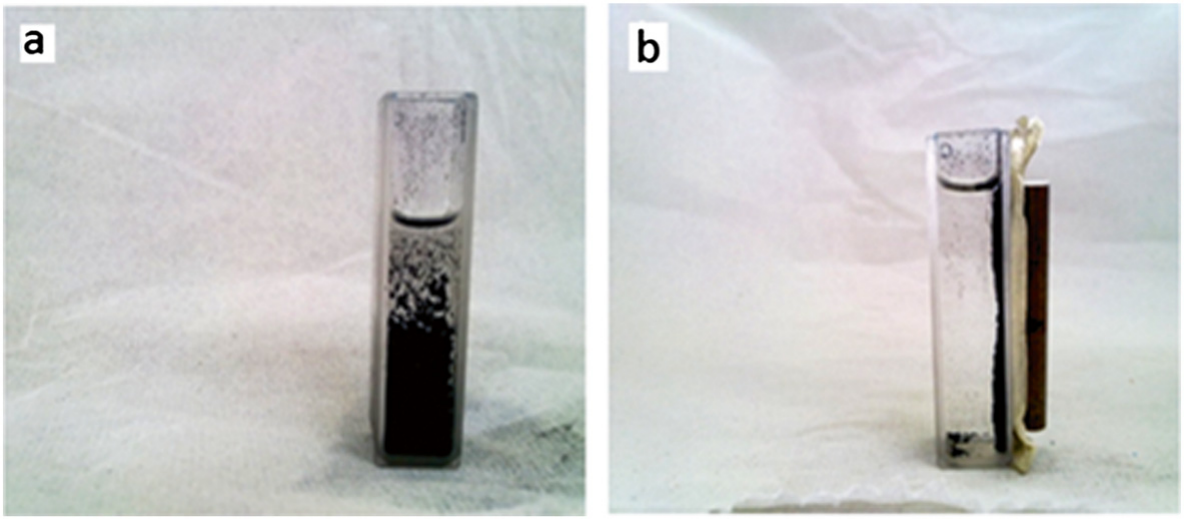
Contaminated magnetic TA-MWCNTs (a) before contact with magnet and (b) after contact with magnet.

Removing of the magnetic TA-MWCNTs after adsorption of boron from aqueous solution was carried out by using a simple magnet. Figure 17 shows the appearance of magnetic TA-MWCNTs in aqueous solution before and after contact with the magnet. As a quantitative analysis, the amount of the remained MWCNTs after contact with magnetic field was measured and the results has shown that only 0.03% of the adsorbent was remained in the solution. For removing the adsorbed boron form TA-MWCNTs, desorption of boron by altering the pH in solution has been suggested by Yu et al. [37]. This will be explored in our future studies.

## Conclusion

The MWCNTs was impregnated with tartaric acid to improve the boron adsorption capacity. The characterization techniques proved forming oxygen functionalities such as carboxylic and phenolic groups on the surface of MWCNTs after modification. Investigation of effective factors on boron capacity of TA-MWCNTs showed that initial solution pH, initial boron concentration, initial adsorbent dosage and temperature had significant effect on boron adsorption. At pH of 6, the maximum adsorption capacity of TA-MWCNTs measured to be 1.97 mg/g under the optimum condition. Also, thermodynamic study showed endothermic, entropy favorable and spontaneous adsorption behavior of boron by TA-MWCNTs. In addition Freundlich model was more suitable to simulate the boron adsorption isotherms than Langmuir model. The TA-MWCNTs was magnetized for separation after adsorption of boron. Magnetic separation of boron contaminated TA-MWCNTs was successfully carried out for further removal from aqueous solution. In conclusion, the modification of MWCNTs with tartaric acid is a successful method for enhancing the adsorption properties of MWCNTs in the removal of boron from aqueous solutions. However, the desorption studies is now of interest and is recommended to be investigated in future works.

## Competing interests

The authors declare that they have no competing interests.

## Authors’ contributions

NZ participated in design of the study, performing the experimental parts, analyzing the data, preparing the report and manuscript. FM participated in design of the study and was involved in preparing the manuscript. LCA participated in design of the study. TSYC participated in design of the study and analyzing the data. All authors read and approved the final manuscript.
